# Prevalence of peripheral neuropathy in pre-diabetes: a systematic review

**DOI:** 10.1136/bmjdrc-2020-002040

**Published:** 2021-05-18

**Authors:** Varo Kirthi, Anugraha Perumbalath, Emily Brown, Sarah Nevitt, Ioannis N Petropoulos, Jamie Burgess, Rebecca Roylance, Daniel J Cuthbertson, Timothy L Jackson, Rayaz A Malik, Uazman Alam

**Affiliations:** 1 Faculty of Life Sciences and Medicine, King’s College London, London, UK; 2 Department of Ophthalmology, King’s College Hospital NHS Foundation Trust, London, UK; 3 Department of Cardiovascular & Metabolic Medicine, Institute of Life Course and Medical Sciences, University of Liverpool, Liverpool, UK; 4 Department of Biostatistics, University of Liverpool, Liverpool, UK; 5 Research Division, Weill Cornell Medicine, Doha, Qatar; 6 Edge Hill University Library, Liverpool University Hospitals NHS Foundation Trust, Liverpool, UK; 7 Division of Cardiovascular Sciences, University of Manchester, Manchester, UK; 8 Division of Endocrinology, Diabetes & Gastroenterology, University of Manchester, Manchester, UK; 9 Pain Research Institute, University of Liverpool, Liverpool, UK

**Keywords:** diabetes complications, diabetic neuropathies, neurology, pre-diabetic state

## Abstract

There is growing evidence of excess peripheral neuropathy in pre-diabetes. We aimed to determine its prevalence, including the impact of diagnostic methodology on prevalence rates, through a systematic review conducted according to Preferred Reporting Items for Systematic Reviews and Meta-Analyses guidelines. A comprehensive electronic bibliographic search was performed in MEDLINE, EMBASE, PubMed, Web of Science and the Cochrane Central Register of Controlled Trials from inception to June 1, 2020. Two reviewers independently selected studies, extracted data and assessed risk of bias. An evaluation was undertaken by method of neuropathy assessment. After screening 1784 abstracts and reviewing 84 full-text records, 29 studies (9351 participants) were included. There was a wide range of prevalence estimates (2%–77%, IQR: 6%–34%), but the majority of studies (n=21, 72%) reported a prevalence ≥10%. The three highest prevalence estimates of 77% (95% CI: 54% to 100%), 71% (95% CI: 55% to 88%) and 66% (95% CI: 53% to 78%) were reported using plantar thermography, multimodal quantitative sensory testing and nerve conduction tests, respectively. In general, studies evaluating small nerve fiber parameters yielded a higher prevalence of peripheral neuropathy. Due to a variety of study populations and methods of assessing neuropathy, there was marked heterogeneity in the prevalence estimates. Most studies reported a higher prevalence of peripheral neuropathy in pre-diabetes, primarily of a small nerve fiber origin, than would be expected in the background population. Given the marked rise in pre-diabetes, further consideration of targeting screening in this population is required. Development of risk-stratification tools may facilitate earlier interventions.

## Introduction

There is an enormous global burden of pre-diabetes, currently estimated to affect 374 million people worldwide and projected to increase to 548 million (8.6% of the global adult population) by 2045.^1^ For pre-diabetes alone, the economic burden in the USA is greater than $43 billion. Pre-diabetes is associated with an increased incidence of diabetes-specific microvascular and macrovascular complications,^2^ and an increase in cardiovascular events and all-cause mortality,^3^ compared with age-matched and body mass index (BMI)-matched people with normal glucose tolerance (NGT). Indeed, the excess risks of a major event (defined as fatal/non-fatal cardiovascular disease or all-cause mortality) were 17% and 12% based on WHO and American Diabetes Association (ADA) criteria, respectively.^3^ End-organ complications of hyperglycemia may therefore be apparent prior to the diagnosis of type 2 diabetes.^4^ Unfortunately, people with pre-diabetes and concomitant microvascular disease are also more likely to develop type 2 diabetes.^5^ Furthermore, the prevalence of diabetic peripheral neuropathy (DPN) has been reported as high as 35% (95% CI: 15% to 55%) at the time of diagnosis of type 2 diabetes, suggestive of an early subclinical disease phase.^6^

In the Toronto international consensus for DPN, a definite diagnosis requires at least one symptom and/or at least one sign of neuropathy and abnormality in nerve conduction studies (NCS).^7^ However, abnormalities in NCS which assess large myelinated nerve fibers that subserve touch, proprioception, vibration and motor function are later manifestations of DPN.^8^ By contrast, small nerve fiber deficits affecting unmyelinated or thinly myelinated C and Aδ nerve fibers involved in thermal, pain and autonomic function are thought to occur much earlier in DPN.^9^ The precise natural history of small and large nerve fiber disease however is still unknown.

Although several studies have reported peripheral neuropathy in pre-diabetes, data in this area are conflicting, with some studies showing a high prevalence of peripheral neuropathy,^10 11^ and others suggesting a low prevalence.^12–14^ In pre-diabetes, continuous or episodic pain is often an early manifestation in the absence of a clinically detectable neuropathy, suggestive of small nerve fiber deficits. Indeed, skin biopsy and corneal confocal microscopy (CCM), both techniques that can quantify early small nerve fiber damage, are abnormal in pre-diabetes.^15 16^ Further evidence of a lack of large fiber involvement is seen in studies using NCS, showing no difference when people with pre-diabetes are compared with those with NGT.^12–14^

In addition to NCS and CCM, several screening and diagnostic tests are available to assess DPN, including physical examination, quantitative sensory testing (QST) and skin biopsy, with a range of sensitivities and specificities.^17^ The National Institute for Health and Care Excellence in the UK recommends vibration perception testing using a 128 Hz tuning fork together with a 10 g Semmes-Weinstein monofilament for the screening of DPN. However, these tests identify advanced DPN at a late, irreversible and pre-ulcerative stage of the diabetic foot.^18^

This systematic review aimed to determine the prevalence of peripheral neuropathy in adults with pre-diabetes and to evaluate how prevalence estimates are influenced by the method of neuropathy assessment. We hypothesise that the diagnostic yield will be determined by the ability of the assessment tool to detect small nerve fiber abnormalities.

## Methods

The protocol for this systematic review was developed using Preferred Reporting Items for Systematic Reviews and Meta-Analyses (PRISMA) guidelines and registered prospectively with the PROSPERO database of systematic reviews (ID: CRD42017080726).

### Search strategy

The following electronic bibliographic databases were searched: MEDLINE (access via OVID), EMBASE (access via OVID), PubMed, Web of Science and the Cochrane Register of Controlled Trials, from inception until June 1, 2020. The search strategy was independently reviewed by an expert information specialist using the Peer Review of Electronic Search Strategies checklist and the MEDLINE search terms are included in online supplemental appendix 1. Additional articles were identified by searching the references of included studies and other review articles identified during the course of the searches. Results from the database searches were merged using an electronic reference manager (EndNote V.X9, Clarivate Analytics, Philadelphia, Pennsylvania, USA) to facilitate removal of duplicates. Relevant publications were retrieved manually from hard copies of journals, if electronic access was not available.

10.1136/bmjdrc-2020-002040.supp1 Supplementary data

### Participants, eligibility and setting

Inclusion criteria were adults over 18 years of age who have pre-diabetes defined either by WHO or ADA criteria. Population-based cohort or cross-sectional studies from any country in any setting were considered, provided they were reported in English and reported prevalence data for peripheral neuropathy. Studies were excluded if they failed to report an independent pre-diabetes group or the prevalence of peripheral neuropathy. Studies exclusively investigating other forms of neuropathy, including autonomic neuropathy, were considered outside the scope of this review and excluded.

### Study selection

Two reviewers independently screened titles and abstracts from the searches. Any disagreements were resolved by discussion with the senior reviewer (UA). All potentially eligible articles were selected for independent full-text assessment by two reviewers. Disagreements between the reviewers were resolved by the senior reviewer (UA), in deciding if an article was eligible for inclusion. A PRISMA flow chart of the selection process is shown in online supplemental appendix 2.

10.1136/bmjdrc-2020-002040.supp2 Supplementary data

### Data collection process

Two reviewers independently extracted data using pre-piloted forms. Data extracted included: (1) date and country of study, (2) study design, (3) age, sex and ethnicity of participants, (4) definition of pre-diabetes, (5) method(s) of assessing peripheral neuropathy, (6) study groups and sizes, (7) overall sample size, and (8) prevalence numbers and estimates.

### Risk of bias assessment

A modified critical appraisal tool specifically developed for assessing risk of bias in prevalence studies was used in all included articles and is included in online supplemental appendix 3.^19^ Quality assessment was conducted independently by two reviewers and any disagreements were resolved by the senior reviewer (UA).

10.1136/bmjdrc-2020-002040.supp3 Supplementary data

### Data analysis

Statistical heterogeneity between included studies was assessed using the I^2^ statistic. Clinical heterogeneity was assessed based on study design, population and methods used to measure peripheral neuropathy. Due to high clinical and statistical heterogeneity (I^2^ >90%), a subsequent meta-analysis was not conducted. Characteristics of included studies and prevalence estimates are presented in summary tables and narrative text. Further evaluation of the included studies was undertaken by comparing methods of neuropathy assessment to assess differences in prevalence estimates.

## Results

### Study and participant characteristics

After removal of duplicate entries, 1526 unique records were identified from the electronic database searches. Titles and abstracts were reviewed against the eligibility criteria and 84 records were selected for full-text review. From these records, 29 studies with 9351 participants fulfilled the eligibility criteria and were included in the final review. Details of the selected articles and reasons for exclusion after full-text review are shown in online supplemental appendix 2. Characteristics of the included studies are presented in Electronic Supplementary Material (ESM) (table 1). Nine of these studies were conducted in single European countries,^16 20–27^ five in the USA,^12–14 28 29^ five in China^30–34^ and two in Japan.^35 36^ One study was conducted across nine countries.^37^ The remaining studies were each conducted in Brazil,^38^ Australia,^39^ Egypt,^40^ India,^41^ Canada,^11^ United Arab Emirates^42^ and Turkey.^43^ Most studies had a cross-sectional population-based or hospital-based design and one was a double-blind randomized controlled trial. The age of included participants varied widely between 20 and 83 years. However, of the 19 studies that reported an average age of included participants, over half (10 of 19, 53%) reported a mean age above 55 years. Gender ratios also varied widely and were reported by the majority of included studies (25 of 29, 86%). Methods of assessing neuropathy and criteria used for defining pre-diabetes are presented in ESM (table 1); 22 studies (76%) used WHO criteria to define pre-diabetes.

**Table 1:** Summary of the methodological quality assessment for each study using the Hoy *et al*^19^ risk of bias tool

Study	Q1	Q2	Q3	Q4	Q5	Q6	Q7	Q8	Q9	Total
Asghar *et al*^16^	1	1	1	1	0	0	0	0	0	4
Balbinot *et al*^38^	0	0	1	1	0	0	0	0	0	2
Barr *et al*^39^	0	0	1	1	0	0	0	0	0	2
Bongaerts *et al*^24^	1	0	1	0	0	0	0	0	0	2
Callaghan *et al*^29^	0	1	1	1	0	0	0	0	0	3
Callaghan *et al*^32^	0	0	0	0	0	0	0	0	1	1
De Neeling *et al*^20^	1	0	0	1	0	0	0	0	0	2
Dimova *et al*^25^	1	0	1	1	0	0	0	0	0	3
Dyck *et al*^14^	0	0	0	1	0	0	0	0	0	1
Franklin *et al*^28^	0	0	0	0	0	0	0	0	0	0
Fujimoto *et al*^12^	1	0	1	1	0	0	0	0	0	3
Fujimoto *et al*^13^	1	0	1	1	0	0	0	0	0	3
Gabriel *et al*^37^	1	0	0	1	0	0	0	0	0	2
Herman *et al*^40^	0	0	0	1	0	0	0	0	0	1
Kannan *et al*^41^	0	1	1	1	0	0	0	0	0	3
Kopf *et al*^27^	1	1	1	0	0	0	0	0	0	3
Kurisu *et al*^36^	0	0	1	1	0	0	0	0	0	2
Lee *et al*^11^	0	1	1	0	0	0	0	0	0	2
Lin *et al*^30^	0	0	0	1	0	0	0	0	0	1
Liu *et al*^33^	1	0	1	1	0	0	0	0	0	3
Lu *et al*^31^	0	1	1	1	0	0	0	0	0	3
Németh *et al*^26^	0	0	1	1	0	0	0	0	0	2
Oohashi *et al*^35^	0	0	1	1	0	0	1	0	0	3
Saadi *et al*^42^	0	0	1	1	0	0	0	0	0	2
Sahin *et al*^43^	0	1	1	1	0	0	0	0	0	3
Zeng *et al*^34^	1	0	1	1	0	0	0	0	0	3
Ziegler *et al*^21^	1	0	0	1	0	0	0	0	0	2
Ziegler *et al*^10^	0	0	0	1	0	0	0	0	0	2
Ziegler *et al*^23^	1	0	0	1	0	0	0	0	0	2

Please see online supplemental appendix 2 for full tool. Q1: Was the study’s target population a close representation of the national population in relation to relevant variables, for example, age, sex, occupation? Q2: Was the sampling frame a true or close representation of the target population? Q3: Was some form of random selection used to select the sample, or was a census undertaken? Q4: Was the likelihood of non-response bias minimal? Q5: Were data collected directly from the subjects (as opposed to a proxy)? Q6: Was an acceptable case definition used in the study? Q7: Was the study instrument that measured the parameter of interest shown to have reliability and validity? Q8: Was the same mode of data collection used for all subjects? Q9: Were the numerator(s) and denominator(s) for the parameter of interest appropriate? Overall risk of bias score: 0–3=low risk, 4–6=moderate risk, 7–9=high risk.

### Risk of bias

Overall quality scores of the reviewed studies and points scored for each item in the critical appraisal tool are provided in table 1. With the exception of Asghar *et al*^16^ which scored 4 (moderate risk of bias), all of the remaining studies were deemed at ‘low’ risk of bias (total score ≤3). The most common issues were: (1) the likelihood of a non-response bias, particularly where participant selection and response rates were not clearly reported (24 studies), (2) failure to undertake a census or use a form of random selection to obtain the sample (19 studies) and (3) the target population of the study not being representative of the national population (12 studies).

### Overall prevalence

**Figure 1:**
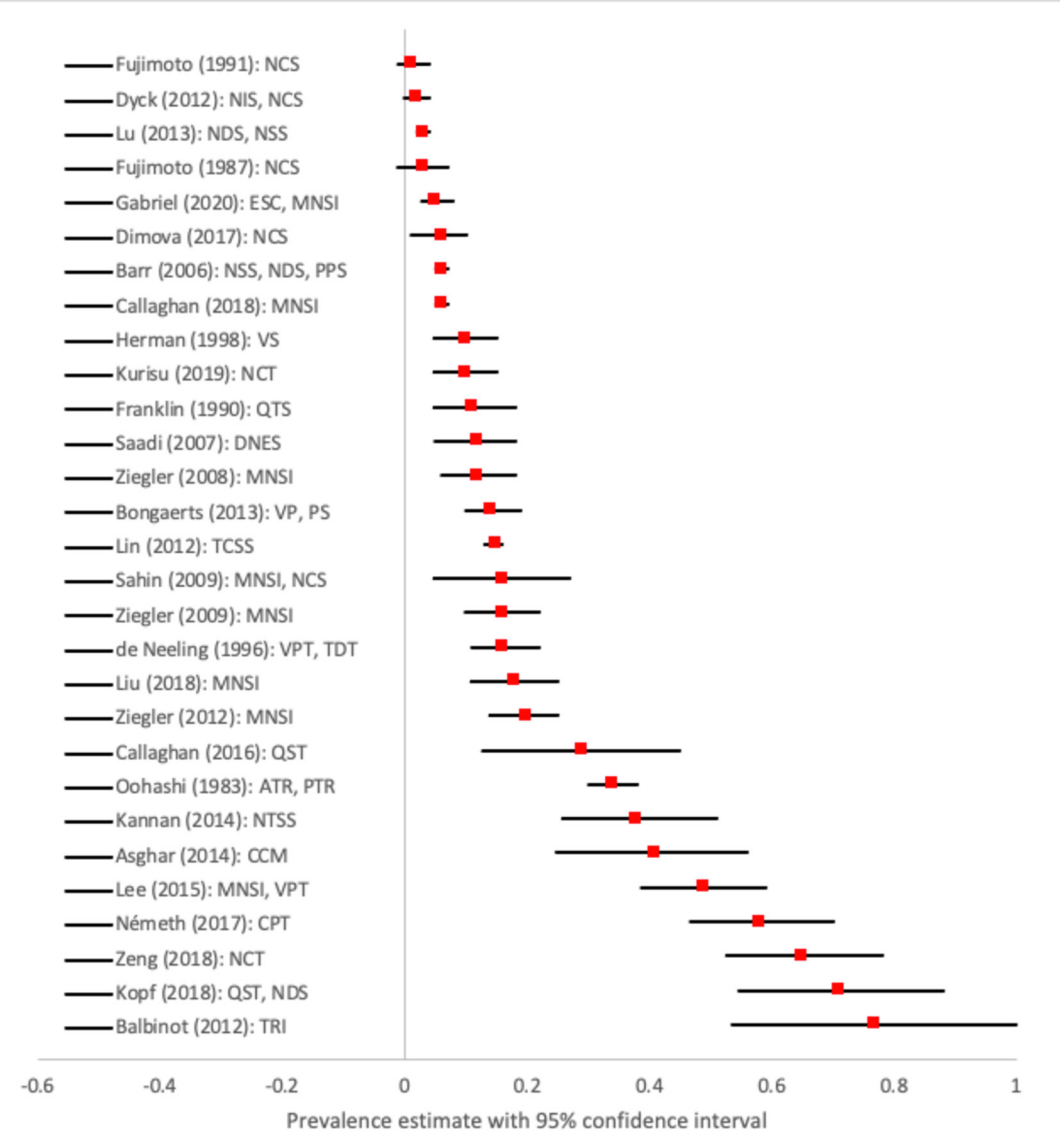
Summary of prevalence estimates of peripheral neuropathy in pre-diabetes for all included studies. Prevalence estimates reported with 95% CIs and primary method used to assess peripheral neuropathy. For three studies (Fujimoto,^12^ Fujimoto,^13^ and Dyck^14^), the 95% confidence intervals around the prevalence estimate were wide and the lower bound of the confidence interval was estimated to be less than 0. This is due to the small numbers used in the estimation of prevalence (in the prevalence group) and the standard error (SE) of prevalence (where SE = square root [p(1-p)/n], where p is the prevalence of peripheral neuropathy as a proportion and n is the total number of people in the study or study group), and the uncertainty of the SE is reflected within the wide confidence intervals. The lower bounds of these negative confidence intervals should be considered to be 0. ATR, Achilles tendon reflex; CCM, corneal confocal microscopy; CPT, current perception threshold; DNES, Diabetic Neuropathy Examination Score; ESC, electrochemical skin conductance; MNSI, Michigan Neuropathy Screening Instrument; NCS, nerve conduction studies; NCT, nerve conduction test; NDS, Neuropathy Disability Score; NIS, Neuropathy Impairment Score; NSS, Neuropathy Symptom Score; NTSS, Neuropathy Total Symptom Score; PPS, Pressure Perception Score; PS, pressure sensation; PTR, patellar tendon reflex; QST, quantitative sensory testing; QTS, quantitative tactile stimulation; TCSS, Toronto Clinical neuropathy Scoring System; TDT, thermal discrimination threshold; TRI, Thermal Recovery Index; VP, vibration perception; VPT, vibration perception threshold; VS, vibration sensation.

Figure 1 shows the prevalence of peripheral neuropathy with 95% CIs for the population with pre-diabetes in each study. Prevalence estimates varied widely, from 2% (95% CI: 0% to 4%) in a study conducted in the USA to 77% (95% CI: 54% to 100%) in a study conducted in Brazil (IQR for all included studies: 6%–34%).^13 38^ The majority of studies (n=21, 72%) reported prevalence estimates ≥10%. Due to a high level of heterogeneity (I^2^ >90%), pooled prevalence estimates were not calculated.

### Methods of assessment

Studies used a combination of clinical histories, questionnaires, physical examinations and quantitative assessments to assess peripheral neuropathy. One study failed to provide information on the method of neuropathy assessment.^35^ The heterogeneity and wide variation in prevalence estimates were in part due to varying methods of neuropathy assessment. Therefore, a narrative summary is presented below.

*Studies using quantitative techniques:* seven studies used quantitative techniques alone to assess peripheral neuropathy.^12–14 20 26 34 40^ Eleven studies presented data from NCS,^12–14 25–27 33 34 36 41 43^ while two measured quantitative vibration sensation.^24 40^ Four studies assessed small nerve fiber function using a variety of methods including CCM, electrochemical skin conductance (ESC), plantar thermography and contact heat pain-evoked potentials.^16 33 37 38^

*NCS:* prevalence estimates for peripheral neuropathy with conventional NCS varied between 2% and 65% using a variety of techniques.^13 34^ However, the four studies that measured nerve conduction parameters alone all reported peripheral neuropathy prevalence estimates of 6% or less.^12–14 25^ Németh *et al*^26^ used the Neurometer R device to evaluate the current perception threshold, a composite of small and large fiber dysfunction, reporting a prevalence estimate of 58%.

*Quantitative vibration sensation:* from the two studies that measured quantitative vibration sensation, prevalence estimates were 14% and 10%, using different methods.^24 40^ In the KORA F4 Study, Bongaerts *et al*^24^ defined peripheral neuropathy as the presence of bilaterally impaired foot-vibration perception and/or foot-pressure sensation, while Herman *et al*^40^ reported a lower prevalence estimate, assessing vibration perception using a forced choice algorithm.

*Quantitative small nerve fiber assessment:* the four studies assessing small nerve fiber function also reported a range of prevalence estimates. Two studies assessed thermal sensation, with Balbinot *et al*^38^ reporting a prevalence of 77% with plantar thermography while Liu *et al*^33^ reported a prevalence of 18% by measuring contact heat pain-evoked potential. CCM demonstrated that 41% of participants with pre-diabetes had evidence of peripheral neuropathy, while ESC showed the prevalence of severe neuropathy, defined as ESC <50 µs in feet or <40 µs in the hands, was 5%.^16 37^

*Studies using physical examinations alone:* two studies used physical examination alone as the primary method of peripheral neuropathy assessment. De Neeling *et al*^20^ used absence of vibration sensation at the big toe as an indicator of peripheral neuropathy, while Saadi *et al*^42^ used the Diabetic Neuropathy Examination Score. The prevalence estimates reported were 16% and 12%, respectively.

*Studies using a combination of methods:* the remaining studies used a combination of questionnaires, physical examination and quantitative assessment. Franklin *et al*^28^ used a combination of history, physical examination and quantitative vibration threshold testing, reporting a peripheral neuropathy prevalence of 11%, which was higher than in participants with NGT (3.9%) but lower than in participants with diabetes (25.8%). Eleven studies used a combination of questionnaires and physical examination. Barr *et al*^39^ and Lu *et al*^31^ used the Neuropathy Symptom Score (NSS) and Neuropathy Disability Score (NDS), while Callaghan *et al*^29^ used the Toronto consensus definition of ‘probable neuropathy’, which requires two or more of neuropathy symptoms, abnormal sensory examination or abnormal reflexes. The Michigan Neuropathy Screening Instrument (MNSI) was the most commonly used scoring tool, detecting a peripheral neuropathy prevalence ranging from 9% to 49% across six studies.^11 21–23 33 43^

### Age, gender and ethnicity

Although age-specific and gender-specific prevalence estimates were not reported, two studies by the same authors separately reported similar prevalence figures in Japanese-American men and women using the same method of assessment.^12 13^ Despite the wide geographic variation in where studies were conducted, none of the included studies reported ethnicity-specific prevalence estimates of peripheral neuropathy.

### Study population

Although estimates of peripheral neuropathy prevalence varied widely between study populations, the vast majority of hospital-based cross-sectional studies reported estimates ≥20% (10 of 12, 83%), in contrast to population-based studies (2 of 15, 13%). The three highest prevalence estimates of 77% (95% CI: 54% to 100%), 71% (95% CI: 55% to 88%) and 65% (95% CI: 53% to 78%) were all reported in hospital-based studies using plantar thermography, long QST and nerve conduction tests, respectively.^27 34 38^ Further analysis by geographic region was limited by differing methods of neuropathy assessment.

### Pre-diabetes subtype

Due to varying methods of assessment, a pooled analysis could not be performed by pre-diabetes subtype. However, four studies presented separate prevalence estimates for impaired fasting glucose (IFG) and impaired glucose tolerance (IGT). In two similar studies defining neuropathy as an MNSI score ≥2, Ziegler *et al*^21 22^ reported a higher prevalence in IGT (13% and 25%) than IFG (11% and 9%, respectively). Based on a Toronto Clinical neuropathy Scoring System score ≥2, Lin *et al*^30^ reported a higher prevalence in combined IFG/IGT (19%) than either isolated IFG (16%) or IGT (13%). Similarly, using vibration perception and perception sensation, Bongaerts *et al*^24^ also reported a higher prevalence of peripheral neuropathy in combined IFG/IGT (24%) than either isolated IFG (6%) or IGT (15%).

## Discussion

### Summary of main results

The majority of studies (21 of 29, 72%) in this systematic review reported a ≥10% prevalence of peripheral neuropathy in pre-diabetes, although with figures varying widely between 2% and 77%, in part due to diagnostic methodology. This is higher than the background prevalence of peripheral neuropathy reported in the general population of 1%–3% (increasing to 7% in the elderly).^44^ Prevalence estimates were consistently higher than participants with NGT, within the same study. From the data evaluated in this review, pre-diabetes is a risk factor for chronic axonal polyneuropathy, consistent with an initial involvement of small nerve fibers. This is a major cause of neuropathic pain and associated morbidity, as well as being an initiating factor in diabetic foot ulceration. Smith and Singleton have suggested that pre-diabetes is common in patients with peripheral neuropathy and that it occurs in approximately 40% of patients with an idiopathic etiology.^45^ Early microvascular complications have been reported by other authors, and our recently published systematic review has also demonstrated an excess of cardiac autonomic neuropathy in pre-diabetes.^46^

### Comparisons with previous data

Understanding the natural history of DPN and peripheral neuropathy in pre-diabetes is vital to determine the optimal screening approach. A systematic review conducted in 2011 found a significant proportion of participants with IGT have mainly small nerve fiber deficits.^47^ In vivo studies support the hypothesis that small nerve fiber deficits are the earliest manifestations of peripheral neuropathy in pre-diabetes.^28 43^ Evidence of early small nerve fiber pathology in IGT also supports this hypothesis. Our study using CCM reported that small nerve fiber deficits predicted progression from IGT to diabetes, and interestingly, improvement of small nerve fiber deficits in participants with IGT who reverted to NGT.^5^ These objectively quantifiable improvements in corneal and intraepidermal nerve morphology that occur in response to changes in glucose tolerance are also in keeping with improvements in large nerve fiber function with metabolic control.^48^

The principal difficulty in appraising the current literature is the variety of diagnostic methodologies used to identify neuropathy, and their relative abilities to assess earlier small nerve fiber versus later large nerve fiber damage. For instance, methods used in annual diabetic foot screening programs (10 g monofilament and 128 Hz tuning fork) have poor sensitivity in the detection of DPN and aim to identify patients at high risk of foot ulceration, hence they do not identify early nerve damage.^49^ While no study solely relied on a questionnaire or patient history to assess peripheral neuropathy, several studies used a combination of screening tools, physical examination and quantitative tests to improve diagnostic sensitivity. For instance, two studies used the NSS questionnaire in combination with physical examination (Lui *et al* and Barr *et al*), with both studies reporting low prevalence rates of peripheral neuropathy. The NSS is thought to poorly reflect the progression of DPN,^50^ despite having a higher diagnostic sensitivity (83%) than the Diabetic Neuropathy Score (64%).^51^ Devigili *et al*^52^ have previously shown that a combination of methods, including clinical abnormalities and small nerve fiber evaluation using QST and skin biopsy, offers a sensitivity of 92.5% for small nerve fiber neuropathy of any cause.

### Small nerve fiber tests for a small nerve fiber disease

Gain of small nerve fiber sensory function has been demonstrated in pre-diabetes. Kopf *et al*^27^ reported a prevalence of mechanical hyperalgesia as high as 33%, although whether this is due to central sensitisation, small nerve fiber dysfunction or a combination of both is debated. However, there is consensus that large nerve fiber involvement only occurs with increasing duration of diabetes, hence there is a paucity of large nerve fiber dysfunction in pre-diabetes, in the early stages of dysglycemia. Dyck *et al*^14^ reported a low prevalence of peripheral neuropathy in IGT similar to individuals with normoglycemia, when performing primarily large nerve fiber diagnostics (NCS). Unfortunately, current consensus endpoints of neuropathy lack sensitivity to capture early small nerve fiber abnormalities prior to the development of overt large nerve fiber pathology. Many of these methods are either invasive (eg, skin biopsy) or have repeatedly failed as surrogate endpoints of therapeutic efficacy in clinical trials of DPN (eg, NCS and QST).

On the contrary, the German Research Network on Neuropathic Pain QST protocol accurately identifies patterns of small nerve fiber deficits.^53^ However, application of the full battery of QST tests is time-consuming and not feasible in routine clinical practice. While skin biopsy is still considered to be the reference standard for the identification of small nerve fiber pathology, mass screening with repeated biopsies is not feasible. CCM, on the other hand, can provide detailed quantification of small nerve fibers and predict the development and progression of DPN.^54 55^ CCM also has increased diagnostic ability when combined with artificial intelligence-based deep learning algorithms and has potential to be implemented on a population basis.^56^

The detection of early peripheral neuropathy in pre-diabetes allows for a multifaceted interventional approach to halt the progression or even reverse neuropathic deficits. Unfortunately, current screening methods detect advanced neuropathy, thus any putative interventions are often ineffective. From these data, NCS alone may not be sufficiently sensitive to identify subclinical peripheral neuropathy in populations with pre-diabetes.^15^

A number of cross-sectional studies have demonstrated the coexistence of small and large nerve fiber abnormalities in DPN.^5 49 57 58^ In the ADDITION Study (Anglo-Danish-Dutch study of Intensive Treatment In peOple with screeN-detected diabetes), CCM could not differentiate the absence or presence of DPN in type 2 diabetes.^59^ Additionally, Ziegler *et al*^60^ reported that nerve fiber loss in recently diagnosed type 2 diabetes, detected by both skin biopsy and CCM, occurred in largely different populations of patients, suggesting a disparate manifestation of small nerve fiber pathology. There was also a reduction in NCS parameters in a subgroup suggestive of large nerve fiber disease.^60^ The precise natural history of DPN remains to be elucidated as at present, the relative contributions and onset of small and large nerve fiber disease remain poorly understood. This is primarily due to a paucity of natural history studies examining the relative contributions of small and large nerve involvement in people at high risk of insulin-resistance states, as they progress from NGT through to pre-diabetes, newly diagnosed diabetes and established diabetes.

Despite these limitations, Asghar *et al*^16^ did show a high prevalence of small nerve fiber pathology (41%) when assessed using CCM, with comparable reductions in intraepidermal nerve fiber density. Future screening and prevalence studies in pre-diabetes should therefore include a validated, reproducible method for detecting small nerve fiber pathology, such as CCM. Importantly, diet and exercise counseling in pre-diabetes results in cutaneous re-inervation and improved pain.^15^

### Prevalence estimates vary by diagnostic tests

The MNSI was used as the primary method of assessment in six of the included studies. Balbinot *et al*^38^ compared MNSI with Thermal Recovery Index, electromyography and interdigital anisothermal technique (IDA). The prevalence of peripheral neuropathy in pre-diabetes was 77%, 15% and 77%, respectively; while MNSI failed to identify peripheral neuropathy in any of the participants, even in cases with an abnormal nerve conduction velocity. IDA, a measure of small nerve fiber dysfunction, was considered the most appropriate test to identify peripheral neuropathy in pre-diabetes by the study authors. Similarly, Sahin *et al*^43^ found the prevalence of peripheral neuropathy to be higher with NCS (21%) than with MNSI (16%). Kopf *et al*^27^ noted the addition of the NDS improved the sensitivity of short QST (testing thermal parameters alone). This supports the argument that a combination of tests improves sensitivity, compared with a single scoring tool or test.

### Prevalence estimates and diagnostic test cut-off values

Another challenge with interpreting prevalence data was the variety of cut-off scores used to define peripheral neuropathy on questionnaires and composite scoring methods. Defining peripheral neuropathy as an MNSI score of ≥2, Ziegler *et al*^23^ reported a prevalence of 31%; however the same study reported a lower prevalence of 20% when peripheral neuropathy was defined as an MNSI score ≥3. The authors also reported a peripheral neuropathy prevalence when using MNSI in combination with a 10 g monofilament test: with an MNSI score ≥2, the prevalence in pre-diabetes was 35% compared with 21% with an MNSI score ≥3. The variability of these prevalence estimates highlights the difficulty in interpreting data even from the same scoring tool.

### Pathophysiology of small nerve fiber deficits in pre-diabetes: IFG, IGT or both?

The etiology of dysglycemia may be of relevance to the development of peripheral neuropathy. In the MONICA/KORA (Cooperative Health Research in the Region Augsburg) Study, the prevalence of peripheral neuropathy was greater in IGT than in participants with NGT.^21^ Out of 195 participants with type 2 diabetes, 71 with IFG, 46 with IGT and 81 with normoglycemia, neuropathic pain was detected in 28%, 11%, 13% and 7%, respectively, which is consistent with preferential small nerve fiber involvement in groups with pre-diabetes.^21^ Bongaerts *et al*^24^ reported similar data with a higher prevalence in IGT (15%), even higher in combined IFG with IGT (24%), when compared with isolated IFG (6%). The annual incidence of type 2 diabetes in individuals with isolated IGT (4%–6%) and isolated IFG (6%–9%) is lower than in those with combined IFG and IGT (15%–19%).^61^ This suggests that isolated IFG and isolated IGT may differ in their pathophysiology, with an IFG–IGT co-diagnosis reflecting a much more severe disturbance of glycemic homeostasis, with a greater risk of progression to type 2 diabetes.

The site of insulin resistance in isolated IFG is predominantly hepatic, whereas people with isolated IGT have greater muscle insulin resistance. Obesity and insulin resistance result in a cascade of metabolic and pro-inflammatory effects which are self-reinforcing and lead to microvascular disease and peripheral nerve injury.^62^ Decreased skeletal muscle sensitivity to insulin leads to adipocytes increasing their uptake of glucose.^62^ This results in the release of free fatty acids and triglycerides, thus resulting in oxidative stress which is a major factor in the development of peripheral nerve injury.^62^ Mitofusin-2 (Mfn2), the gene responsible for Charcot-Marie-Tooth type 2A, encodes a mitochondrial protein that regulates mitochondrial metabolism and intracellular signaling. Mfn2 mRNA is downregulated in type 2 diabetes, upregulated in weight loss and is inversely proportional to BMI in skeletal muscle.^63^ Mfn2 mutations are known to cause severe phenotypes of neuropathy in Charcot-Marie-Tooth type 2A.^64^

### Limitations and future work

A number of methodological issues were identified, including the wide variability of sample size. Only four studies had pre-diabetes sample sizes greater than 1000 participants^30–32 39^; the remainder were smaller, with the smallest reporting only 13 participants.^38^ Such small studies are at particular risk of reporting bias and overestimating the magnitude of peripheral neuropathy prevalence. Population bias also limited the generalisability of the prevalence data; many studies recruited participants from hospital clinics, which may not accurately represent the burden of pre-diabetes within the general population. We only included published data and limited studies to those in English language only. Due to a high level of clinical heterogeneity from the variety of diagnostic approaches used, and statistical heterogeneity from variations in study design and sampling methods, a meta-analysis could not be performed. Many studies failed to report prevalence figures or reported the prevalence of pre-diabetes in individuals with peripheral neuropathy, therefore failing to meet the inclusion criteria for this review. Given the significant excess of peripheral neuropathy noted in the included studies, large prospective population-based and mechanistic studies are now required. Such studies should use standardized quantitative methods, evaluating small nerve fibers to accurately determine the prevalence of peripheral neuropathy and accurately delineate its pathophysiology. Future research includes the development of risk-stratification tools to identify those most at risk of peripheral neuropathy in pre-diabetes, to ensure the feasibility of any proposed screening methods.

## Conclusions

This systematic review reports a high prevalence of a primary axonal polyneuropathy in pre-diabetes. There is a need to develop risk-stratification tools to identify those most at risk of peripheral neuropathy. Future clinical trials are needed to explore the potential benefits of early interventions with novel pharmacotherapy, dietary and weight loss interventions, such as low-calorie diets and multifactorial risk factor modification, in this at-risk population.

10.1136/bmjdrc-2020-002040.supp4 Supplementary data

## Data Availability

All data relevant to the study are included in the article or uploaded as supplemental information. This systematic review summarizes publicly available published literature.
