# Supplementary material for: Prevalence of peripheral neuropathy in pre-diabetes: a systematic review
Source: BMJ Open Diabetes Res Care. 2021 May 18;9(1):e002040. doi: 10.1136/bmjdrc-2020-002040 (PMC8137250; doi:10.1136/bmjdrc-2020-002040)
Supplement: Supplementary data [file bmjdrc-2020-002040supp001.pdf]

APPENDIX 1 – MEDLINE SEARCH TERMS

Search terms:

1) impaired glucose tolerance
2) impaired fasting tolerance
3) prediabetes
4) pre-diabetes
5) 1 or 2 or 3 or 4
6) neuropathy
7) peripheral neuropathy
8) small fibre neuropathy
9) polyneuropathy
10) painful neuropathy
11) pain
12) distal symmetrical polyneuropathy
13) idiopathic small fibre neuropathy
14) painful diabetic neuropathy
15) 6 or 7 or 8 or 9 or 10 or 11 or 12 or 13 or 14
16) prevalence
17) occurrence
18) incidence
19) burden
20) epidemiology
21) frequency
22) 16 or 17 or 18 or 19 or 20 or 21
23) 5 and 15 and 22
