# Supplementary material for: Prevalence of peripheral neuropathy in pre-diabetes: a systematic review
Source: BMJ Open Diabetes Res Care. 2021 May 18;9(1):e002040. doi: 10.1136/bmjdrc-2020-002040 (PMC8137250; doi:10.1136/bmjdrc-2020-002040)
Supplement: Supplementary data [file bmjdrc-2020-002040supp002.pdf]

APPENDIX 2 – PRISMA FLOW DIAGRAM

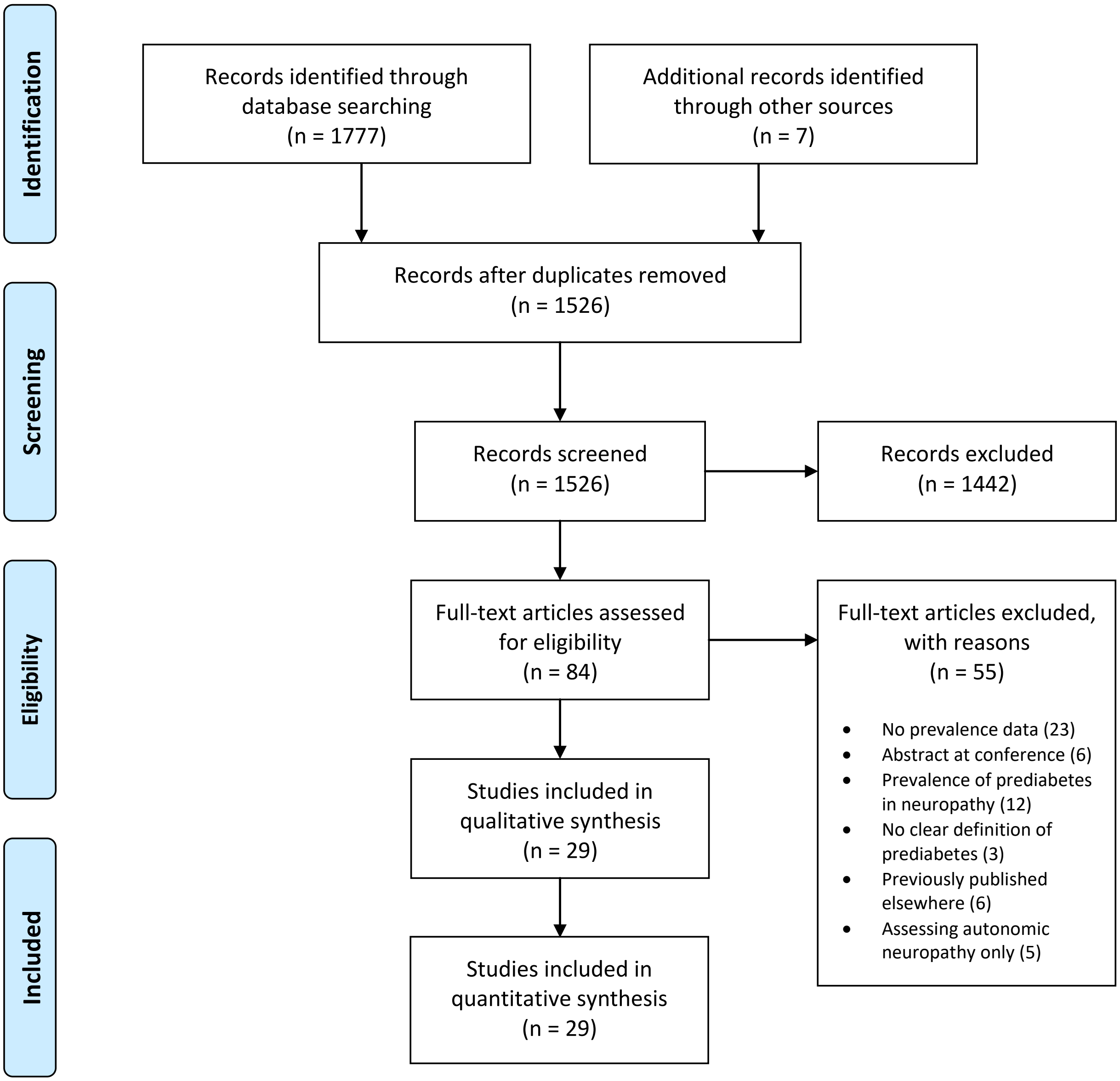

From: Moher D, Liberati A, Tetzlaff J, Altman DG, The PRISMA Group (2009). *P*referred *R*eporting *I*tems for *S*ystematic Reviews and *M*eta-*A*nalyses: The PRISMA Statement. PLoS Med 6(7): e1000097. doi:10.1371/journal.pmed1000097

For more information, visit www.prisma-statement.org.
