# Supplementary material for: Prevalence of peripheral neuropathy in pre-diabetes: a systematic review
Source: BMJ Open Diabetes Res Care. 2021 May 18;9(1):e002040. doi: 10.1136/bmjdrc-2020-002040 (PMC8137250; doi:10.1136/bmjdrc-2020-002040)
Supplement: Supplementary data [file bmjdrc-2020-002040supp003.pdf]

APPENDIX 3 – CRITICAL APPRAISAL TOOL FOR PREVALENCE STUDIES BY HOY *ET AL*. (2012)*

Name of author(s):
Year of publication:
Study title:

**Risk of bias items**		**Risk of bias levels**	**Points scored**
1	Was the study's target population a close representation of the national population in relation to relevant variables, e.g. age, sex, occupation?	**Yes (LOW RISK):** The study's target population was a close representation of the national population.	**0**
		**No (HIGH RISK):** The study's target population was clearly NOT representative of the national population.	**1**
2	Was the sampling frame a true or close representation of the target population?	**Yes (LOW RISK):** The sampling frame was a true or close representation of the target population.	**0**
		**No (HIGH RISK):** The sampling frame was NOT a true or close representation of the target population.	**1**
3	Was some form of random selection used to select the sample, OR, was a census undertaken?	**Yes (LOW RISK):** A census was undertaken, OR, some form of random selection was used to select the sample (e.g. simple random sampling, stratified random sampling, cluster sampling, systematic sampling).	**0**
		**No (HIGH RISK):** A census was NOT undertaken, AND some form of random selection was NOT used to select the sample.	**1**
4	Was the likelihood of non-response bias minimal?	**Yes (LOW RISK):** The response rate for the study was ≥75%, OR, an analysis was performed that showed no significant difference in relevant demographic characteristics between responders and non- responders.	**0**
		**No (HIGH RISK):** The response rate was <75%, and if any analysis comparing responders and non-responders was done, it showed a significant difference in relevant demographic characteristics between responders and non-responders.	**1**
5	Were data collected directly from the subjects (as opposed to a proxy)?	**Yes (LOW RISK):** All data were collected directly from the subjects.	**0**
		**No (HIGH RISK):** In some instances, data were collected from a proxy.	**1**
6	Was an acceptable case definition used in the study?	**Yes (LOW RISK):** An acceptable case definition was used.	**0**
		**No (HIGH RISK):** An acceptable case definition was NOT used.	**1**

7	Was the study instrument that measured the parameter of interest (e.g. prevalence of low back pain) shown to have reliability and validity (if necessary)?	**Yes (LOW RISK):** The study instrument had been shown to have reliability and validity (if this was necessary), e.g. test-re- test, piloting, validation in a previous study, etc.	**0**
		**No (HIGH RISK):** The study instrument had NOT been shown to have reliability or validity (if this was necessary).	**1**
8	Was the same mode of data collection used for all subjects?	**Yes (LOW RISK):** The same mode of data collection was used for all subjects.	**0**
		**No (HIGH RISK):** The same mode of data collection was NOT used for all subjects.	**1**
9	Were the numerator(s) and denominator(s) for the parameter of interest appropriate?	**Yes (LOW RISK):** The paper presented appropriate numerator(s) AND denominator(s) for the parameter of interest (e.g. the prevalence of low back pain).	**0**
		**No (HIGH RISK):** The paper did present numerator(s) AND denominator(s) for the parameter of interest but one or more of these were inappropriate.	**1**
10	Summary on the overall risk of study bias	**LOW RISK**	**0-3**
		**MODERATE RISK**	**4-6**
		**HIGH RISK**	**7-9**

* **Reference:** Hoy D, Brooks P, Woolf A, Blyth F, March L, Bain C, et al. Assessing risk of bias in prevalence studies: modification of an existing tool and evidence of interrater agreement. J Clin Epidemiol. 2012 Sep;65(9):934–9.
