# Supplementary material for: Prevalence of peripheral neuropathy in pre-diabetes: a systematic review
Source: BMJ Open Diabetes Res Care. 2021 May 18;9(1):e002040. doi: 10.1136/bmjdrc-2020-002040 (PMC8137250; doi:10.1136/bmjdrc-2020-002040)
Supplement: Supplementary data [file bmjdrc-2020-002040supp004.pdf]

ELECTRONIC SUPPLEMENTARY MATERIAL

ESM Table 1. Summary of data extraction

**Author**	**Country**	**Study design**	**Method(s) of neuropathy assessment used**	**Primary method of assessment**	**Pre-diabetes definition**	**Total sample size**	**Study groups** (subgroups by method)	**Age in years** (range or mean ± SD)	**Gender** (M/F)	**Sample size**	**Participants affected, *n***	**Prevalence estimate** (%)
Asghar *et al*. (2014)	United Kingdom	Cross-sectional, hospital-based	Neuropathy symptom profile, McGill pain index, NCS, QST, skin biopsy, heart rate variability, deep breathing, skin biopsy and CCM	Corneal nerve fibre density ≤24.0 no/mm^2 on CCM (using manual CCMetrics analysis)	WHO	57	IGT<br>NGT	30-75	NR	37<br>20	15<br>NR	41<br>NR
Balbinot *et al*. (2012)	Brazil	Cross-sectional, hospital-based	MNSI, TRI, EMG, IDA, HRV	MNSI	ADA	79	**MNSI:**<br>DM<br>Pre-DM<br>Control<br><br>**TRI:**<br>DM<br>Pre-DM<br>Control<br><br>**EMG:**<br>DM<br>Pre-DM<br>Control<br><br>**IDA:**<br>DM<br>Pre-DM<br>Control	 55.9 ± 9.4<br>56.8 ± 12.6<br>45.1 ±14.9<br><br><br>55.9 ± 9.4<br>56.8 ± 12.6<br>45.1 ±14.9<br><br><br>55.9 ± 9.4<br>56.8 ± 12.6<br>45.1 ±14.9<br><br><br>55.9 ± 9.4<br>56.8 ± 12.6<br>45.1 ±14.9	 9/20<br>3/10<br>16/21<br><br><br>9/20<br>3/10<br>16/21<br><br><br>9/20<br>3/10<br>16/21<br><br><br>9/20<br>3/10<br>16/21	 29<br>13<br>37<br><br><br>29<br>13<br>37<br><br><br>29<br>13<br>37<br><br><br>29<br>13<br>37	 2<br>0<br>0<br><br><br>22<br>10<br>14<br><br><br>16<br>2<br>0<br><br><br>20<br>10<br>13	 7<br>0<br>0<br><br><br>76<br>77<br>38<br><br><br>55<br>15<br>0<br><br><br>69<br>77<br>35
Barr *et al*. (2006)	Australia	Cross-sectional, population-based	Modified NSS, modified NDS, PPS and postural systolic blood pressure drop score >20mmHg	Neuropathy if ≥2 methods were abnormal	WHO	1154	IFG/IGT	≥25 years	NR	1084	66	6

**Author**	**Country**	**Study design**	**Method(s) of neuropathy assessment used**	**Primary method of assessment**	**Pre-diabetes definition**	**Total sample size**	**Study groups** (subgroups by method)	**Age in years** (range or mean ± SD)	**Gender** (M/F)	**Sample size**	**Participants affected, *n***	**Prevalence estimate** (%)
Bongaerts *et al.* (2013)	Germany	Cross-sectional, population-based	Interview addressing pain, presence of pain or neurological disease and history of foot ulcers or amputation; foot inspection and neurological tests of touch, vibration and temperature; ankle reflexes and sudomotor function	Vibration perception and pressure sensation	WHO	1100	*DM (total)*<br>Known DM<br>Newly diagnosed DM<br>*Pre-DM (total)*<br>i-IFG<br>i-IGT<br>IFG-IGT<br>NGT	61-82	NR	*239*<br>177<br>62<br>*284*<br>55<br>183<br>46<br>577	*49*<br>39<br>10<br>*41*<br>3<br>27<br>11<br>64	*21*<br>22<br>16<br>*14*<br>6<br>15<br>24<br>11
Callaghan *et al.* (2016)	United States	Cross-sectional, hospital-based	Quantitative sudomotor axon reflex testing, QST, Neuro-QoL and the short-form McGill pain questionnaire	Toronto consensus definition of probable polyneuropathy	ADA	155	DM (obese)<br>Pre-DM (obese)<br>NGT (obese)<br>NGT (lean)	52.9 ± 10.2 (obese total)<br><br>48.5 ± 9.9	48/54 (obese total)<br>16/37	26<br>31<br>45<br>53	9<br>9<br>5<br>2	35<br>29<br>11<br>4
Callaghan *et al.* (2018)	China	Cross-sectional, population-based	MNSI questionnaire, MNSI testing and monofilament testing	MNSI ≥2.5	ADA	4002	**Overall:**<br>DM<br>Pre-DM<br>NGT<br><br>**MNSI questionnaire:**<br>DM<br>Pre-DM<br>NGT<br><br>**Monofilament:**<br>DM<br>Pre-DM<br>NGT<br><br>**MNSI + monofilament:**<br>DM<br>Pre-DM<br>NGT	 56.7 ± 9.9<br>53.2 ± 11.3<br>47.2 ± 11.8<br><br><br>NR<br><br><br><br><br>NR<br><br><br><br><br>NR	 338/419<br>816/942<br>885/602<br><br><br>NR<br><br><br><br><br>NR<br><br><br><br><br>NR	 757<br>1758<br>1487<br><br><br>NR<br><br><br><br><br>NR<br><br><br><br><br>NR	 NR	 NR<br><br><br><br><br>6<br>2<br>1<br><br><br>9<br>7<br>4<br><br><br>15<br>6<br>3
de Neeling *et al.* (1996)	Netherlands	Cross-sectional, population-based	VPT and TDT	Vibration sensation absent at big toe	WHO	597	Known DM<br>Newly-diagnosed DM<br>IGT<br>NGT	65.9 ± 6.8<br>65.9 ± 6.6<br>65.2 ± 6.9<br>63.3 ± 7.4	30/43<br>42/48<br>78/89<br>134/133	73<br>90<br>167<br>267	50<br>39<br>27<br>28	69<br>43<br>16<br>11

**Author**	**Country**	**Study design**	**Method(s) of neuropathy assessment used**	**Primary method of assessment**	**Pre-diabetes definition**	**Total sample size**	**Study groups** (subgroups by method)	**Age in years** (range or mean ± SD)	**Gender** (M/F)	**Sample size**	**Participants affected, *n***	**Prevalence estimate** (%)
Dimova *et al.* (2017)	Bulgaria	Cross-sectional, hospital-based	Abnormal NCS and presence of symptoms or signs of neuropathy	Abnormal NCS and presence of symptoms or signs of neuropathy	WHO	478	DM<br>IFG<br>IGT<br>NGT	54.4 ± 11.5<br>51.7 ± 12.1<br>49.2 ± 13.5<br>46.6 ± 11.4	54/67<br>71/54<br>71/31<br>82/48	121<br>125<br>102<br>130	NR	29<br>6 (pre-DM total)<br>0
Dyck *et al.* (2012)	United States	Cross-sectional, population-based	NIS and NCS	Composite scores of nerve conduction - ∑ 2NC ≤2.5th percentile	ADA	542	New DM<br>IGT<br>NGT	23-76<br>22-76<br>25-76	132/86<br>118/56<br>81/69	218<br>174<br>150	17<br>3<br>3	8<br>2<br>2
Franklin *et al.* (1990)	United States	Case control	History and neurological examination, QTS	2 of 3 criteria for definite neuropathy	WHO	852	DM<br>Pre-DM<br>NGT	20-74	NR	277<br>89<br>486	77<br>10<br>17	28<br>11<br>4
Fujimoto *et al.* (1987)	United States	Cross-sectional, population-based	NCS	NCV or distal latency >2 SD above mean in 3 nerves	WHO	229	DM<br>IGT<br>NGT	NR	All male	78<br>72<br>79	36<br>2<br>4	46<br>3<br>5
Fujimoto *et al.* (1991)	United States	Cross-sectional, population-based	NCS	NCV or distal latency >2 SD above mean in 3 nerves	WHO	191	DM<br>IGT<br>NGT	63.7 ± 6.2<br>62.7 ± 6.4<br>61.9 ± 5.9	All female	52<br>67<br>72	4<br>1<br>0	8<br>2<br>0
Gabriel *et al.* (2020)	Australia, Austria, Bulgaria, Greece, Kuwait, Poland, Serbia, Spain and Turkey	Double-blind, placebo-controlled randomised controlled trial	SUDOSCAN (ESC) and MNSI questionnaire	Severe neuropathy according to electrochemical skin conductance on SUDOSCAN (<50µS feet or <40µS hands)	WHO	809	Pre-DM	45-74	339/470	809	43	5

**Author**	**Country**	**Study design**	**Method(s) of neuropathy assessment used**	**Primary method of assessment**	**Pre-diabetes definition**	**Total sample size**	**Study groups** (subgroups by method)	**Age in years** (range or mean ± SD)	**Gender** (M/F)	**Sample size**	**Participants affected, *n***	**Prevalence estimate** (%)
Herman *et al*. (1998)	Egypt	Cross-sectional, population-based	Foot examination and quantitative assessment of vibratory sensation	Vibration sensation ≥ 4 SD above non-DM population aged 20-44	WHO	1451	DM<br>Undiagnosed DM<br>IGT<br>NGT	>20	135/249<br>39/86<br>58/82<br>328/427	384<br>125<br>140<br>750	84<br>17<br>14<br>46	22<br>14<br>10<br>6
Kannan *et al*. (2014)	India	Cross-sectional, hospital-based	Clinical examination using NTSS and NCS	NTSS	WHO	88	IGT<br>NGT	35-72 (total)<br>53.8 mean	42/16	58<br>30	19<br>NR	38<br>NR
Kopf *et al*. (2018)	Germany	Cross-sectional, hospital-based	NDS, NSS, NCS, short QST and long QST	Long QST	WHO	136	**Long QST:**<br>DM<br>Pre-DM<br><br>**Long QST + NDS:**<br>DM<br>Pre-DM<br><br>**Short QST:**<br>DM<br>Pre-DM<br><br>**Short QST + NDS:**<br>DM<br>Pre-DM<br><br>**NDS:**<br>DM<br>Pre-DM	 64.9 ± 2.0<br>60.0 ± 3.0<br><br><br>64.9 ± 2.0<br>60.0 ± 3.0<br><br><br>64.9 ± 2.0<br>60.0 ± 3.0<br><br><br>64.9 ± 2.0<br>60.0 ± 3.0<br><br><br>64.9 ± 2.0<br>60.0 ± 3.0	 67/41<br>13/15<br><br><br>67/41<br>13/15<br><br><br>67/41<br>13/15<br><br><br>67/41<br>13/15<br><br><br>67/41<br>13/15	 108<br>28<br><br><br>108<br>28<br><br><br>108<br>28<br><br><br>108<br>28<br><br><br>108<br>28	 103<br>20<br><br><br>103<br>20<br><br><br>53<br>10<br><br><br>81<br>12<br><br><br>68<br>3	 95<br>71<br><br><br>95<br>71<br><br><br>49<br>36<br><br><br>75<br>43<br><br><br>63<br>11

**Author**	**Country**	**Study design**	**Method(s) of neuropathy assessment used**	**Primary method of assessment**	**Pre-diabetes definition**	**Total sample size**	**Study groups** (subgroups by method)	**Age in years** (range or mean ± SD)	**Gender** (M/F)	**Sample size**	**Participants affected, *n***	**Prevalence estimate** (%)
Kurisu *et al.* (2019)	Japan	Cross-sectional, population-based	Toronto consensus, ATR, QVT, sural nerve amplitude and conduction velocity on DPNCheck device	NCT on DPNCheck	WHO	625	**NCT on DPNCheck:**					
							Known DM	66.1 ± 6.2	39/23	62	15	24
							New DM	66.4 ± 5.6	5/8	13	3	23
							Pre-DM	65.2 ± 7.6	57/63	120	12	10
							NGT	60.4 ± 9.9	159/271	430	31	7
							**Diminished ATR:**					
							Known DM	66.1 ± 6.2	39/23	62	19	31
							New DM	66.4 ± 5.6	5/8	13	0	0
							Pre-DM	65.2 ± 7.6	57/63	120	12	10
							NGT	60.4 ± 9.9	159/271	430	37	9
							**Abnormal QVT:**					
							Known DM	66.1 ± 6.2	39/23	62	4	7
							New DM	66.4 ± 5.6	5/8	13	0	0
							Pre-DM	65.2 ± 7.6	57/63	120	2	2
							NGT	NR	NR	429	13	3
							**Abnormal AMP:**					
							Known DM	66.1 ± 6.2	39/23	62	9	15
							New DM	66.4 ± 5.6	5/8	13	2	15
							Pre-DM	65.2 ± 7.6	57/63	120	7	6
							NGT	NR	NR	426	14	3
							**Abnormal CV:**					
							Known DM	66.1 ± 6.2	39/23	62	9	15
							New DM	66.4 ± 5.6	5/8	13	1	8
							Pre-DM	65.2 ± 7.6	57/63	120	6	5
							NGT	NR	NR	429	19	5
							**NCA:**					
							Known DM	66.1 ± 6.2	39/23	62	15	24
							New DM	66.4 ± 5.6	5/8	13	3	23
							Pre-DM	65.2 ± 7.6	57/63	120	12	10
							NGT	NR	NR	429	31	7

**Author**	**Country**	**Study design**	**Method(s) of neuropathy assessment used**	**Primary method of assessment**	**Pre-diabetes definition**	**Total sample size**	**Study groups** (subgroups by method)	**Age in years** (range or mean ± SD)	**Gender** (M/F)	**Sample size**	**Participants affected, *n***	**Prevalence estimate** (%)
Lee *et al.* (2015)	Canada	Cross-sectional, hospital-based	MNSI and VPT	Neuropathy defined by MNSI ≥2	WHO	467	DM<br>Pre-DM<br>NGT/NFG	53 ± 7	126/341	22<br>101<br>344	11<br>50<br>100	50<br>49<br>29
Lin *et al.* (2012)	China	Cross-sectional, population-based	TCSS	TCSS ≥6	WHO	5385	DM<br>IFG<br>IGT<br>IFG & IGT<br>NGT	54.5 ± 10.9<br>49.2 ± 11.9<br>50.5 ± 11.4<br>52.0 ± 11.4<br>44 ± 13.7	212/210<br>75/200<br>439/611<br>78/213<br>1503/1844	422<br>275<br>1050<br>291<br>3347	120<br>44<br>138<br>54<br>321	28<br>16<br>13<br>19<br>10
Liu *et al.* (2018)	China	Cross-sectional, hospital-based	NCS, SSR, CHEP and MNSI	MNSI >2	WHO	180	Pre-DM<br>NGT	35-81	84/96	120<br>60	22<br>NR	18<br>NR
Lu *et al.* (2013)	China	Cross-sectional, population-based	NDS and NSS	Symptoms and signs of neuropathy; NDS ≥6 with no symptoms; mild signs with moderate symptoms, NDS ≥3 and NSS ≥5	ADA	2035	DM<br>Pre-DM<br>NGT	64.0 ± 9.9<br>61.0 ± 9.4<br>59.7 ± 11.2	230/304<br>357/686<br>141/317	534<br>1043<br>458	43<br>29<br>7	8<br>3<br>2
Németh *et al.* (2017)	Hungary	Cross-sectional, hospital-based	CPT of median and peroneal nerves using Neurometer R device	≥2 abnormal readings on any frequencies on the upper or lower limbs	WHO	111	IGT<br>NGT	58.7 ± 11.1<br>55.1 ± 10.1	33/39<br>17/22	72<br>39	42<br>4	58<br>10
Oohashi *et al.* (1983)	Japan	Cross-sectional, hospital-based	ATR, PTR	†<br>‡	WHO	3809	DM – A<br>DM – B<br>IGT	>25	2179/1630	2912<br>334<br>563	1835<br>157<br>191	63<br>47<br>34

**Author**	**Country**	**Study design**	**Method(s) of neuropathy assessment used**	**Primary method of assessment**	**Pre-diabetes definition**	**Total sample size**	**Study groups** (subgroups by method)	**Age in years** (range or mean ± SD)	**Gender** (M/F)	**Sample size**	**Participants affected, *n***	**Prevalence estimate** (%)
Saadi *et al.* (2007)	United Arab Emirates	Cross-sectional, population-based	DNES	DNES ‡	WHO	373	Diagnosed DM<br>Undiagnosed DM<br>Pre-DM<br>NGT	56.0 ± 11.6<br>49.9 ± 11.8<br>41.4 ± 14.3<br>34.6 ± 13.7	23/34<br>10/30<br>24/61<br>65/126	57<br>40<br>85<br>191	20<br>6<br>10<br>NR	35<br>16<br>12<br>NR
Sahin *et al.* (2009)	Turkey	Cross sectional, hospital-based	MNSI and NCS	MNSI and NCS	ADA	77	**MNSI:**<br>Pre-DM<br>NGT<br><br>**NCS:**<br>Pre-DM	 53 ± 12<br>50 ± 15<br><br><br>53 ± 12	 13/30<br>6/28<br><br><br>13/30	 43<br>34<br><br><br>43	 7<br>NR<br><br><br>9	 16<br>NR<br><br><br>21
Zeng *et al.* (2018)	China	Cross-sectional, hospital-based	Dorsal-sural and medial-plantar SNAP, NCT, NDS and NSS	NCT	§	158	**NCT:**<br>DM<br>Pre-DM<br>NGT<br><br>**Dorsal-sural SNAP:**<br>DM<br>Pre-DM<br>NGT<br><br>**Dorsal-sural NCV:**<br>DM<br>Pre-DM<br>NGT<br><br>**Medial-plantar SNAP:**<br>DM<br>Pre-DM<br>NGT<br><br>**Medial-plantar NCV:**<br>DM<br>Pre-DM<br>NGT	 51.6 ± 6.9<br>51.9 ± 8.7<br>51.8 ± 8.8<br><br><br>51.6 ± 6.9<br>51.9 ± 8.7<br>51.8 ± 8.8<br><br><br>51.6 ± 6.9<br>51.9 ± 8.7<br>51.8 ± 8.8<br><br><br>51.6 ± 6.9<br>51.9 ± 8.7<br>51.8 ± 8.8<br><br><br>51.6 ± 6.9<br>51.9 ± 8.7<br>51.8 ± 8.8	 26/29<br>19/36<br>22/26<br><br><br>26/29<br>19/36<br>22/26<br><br><br>26/29<br>19/36<br>22/26<br><br><br>26/29<br>19/36<br>22/26<br><br><br>26/29<br>19/36<br>22/26	 55<br>55<br>48<br><br><br>55<br>55<br>48<br><br><br>55<br>55<br>48<br><br><br>55<br>55<br>48<br><br><br>55<br>55<br>48	 42<br>36<br>0<br><br><br>40<br>35<br>0<br><br><br>42<br>36<br>0<br><br><br>45<br>34<br>2<br><br><br>47<br>35<br>5	 76<br>65<br>0<br><br><br>73<br>64<br>0<br><br><br>76<br>66<br>0<br><br><br>82<br>62<br>4<br><br><br>86<br>64<br>10

**Author**	**Country**	**Study design**	**Method(s) of neuropathy assessment used**	**Primary method of assessment**	**Pre-diabetes definition**	**Total sample size**	**Study groups** (subgroups by method)	**Age in years** (range or mean ± SD)	**Gender** (M/F)	**Sample size**	**Participants affected, *n***	**Prevalence estimate** (%)
Ziegler *et al*. (2008)	Germany	Cross-sectional, population-based	MNSI	Neuropathy defined by MNSI ≥2	WHO	393	DM<br>IFG<br>IGT<br>NGT	66.8 ± 9.4<br>66.6 ± 8.1<br>69.3 ± 7.8<br>63.6 ± 9.3	110/85<br>45/26<br>23/23<br>43/38	195<br>71<br>46<br>81	NR	28<br>11<br>13<br>7
Ziegler *et al*. (2009)	Germany	Cross-sectional, population-based	MNSI	Neuropathy defined by MNSI ≥2	WHO	426	DM<br>IFG<br>IGT<br>NGT	69.0 ± 8.1<br>72.0 ± 6.8<br>70.9 ± 6.8<br>71.1 ± 6.3	172/42<br>56/14<br>44/17<br>59/22	214<br>70<br>61<br>81	NR	39<br>9<br>25<br>17
Ziegler *et al*. (2012)	Germany	Cross-sectional, population-based	MNSI	Neuropathy defined by MNSI ≥2	WHO	940	DM<br>Pre-DM	71.8 ± 5.5<br>71.5 ± 5.2	117/84<br>124/107	201<br>231	60<br>45	30<br>20

Footnotes: ***ADA***: *American Diabetes Association,* ***ATR***: *Achilles tendon reflex,* ***CCM***: *corneal confocal microscopy,* ***CHEP***: *contact heat pain evoked potential,* ***CPT***: *current perception threshold,* ***DM***: *diabetes mellitus,* ***DNES***: *Diabetic Neuropathy Examination Score,* ***EMG***: *electromyography,* ***ESC***: *electrochemical skin conductance,* ***HRV***: *heart rate variability,* ***IDA***: *interdigital anisothermal technique,* ***i-IGT***: *isolated impaired glucose tolerance,* ***IGT***: *impaired glucose tolerance,* ***i-IFG***: *isolated impaired fasting glucose,* ***IFG***: *impaired fasting glucose,* ***MNSI***: *Michigan Neuropathy Screening Instrument,* ***NCS***: *nerve conduction studies,* ***NCT***: *nerve conduction tests,* ***NCV***: *nerve conduction velocity,* ***NDS***: *neuropathy disability score,* ***NIS***: *neuropathy impairment score,* ***Neuro-QoL***: *neuropathy-specific quality of life in neurological disorders instrument,* ***NFG***: *normal fasting glucose,* ***NGT***: *normal glucose tolerance,* ***NSS***: *neuropathy symptom score,* ***NTSS***: *neuropathy total symptom score,* ***NR***: *numerical data not reported,* ***OTS***: *Optacon tactile stimulator,* ***Pre-DM***: *pre-diabetes,* ***PPS***: *pressure perception score,* ***PTR***: *patellar tendon reflex,* ***QST***: *quantitative sensory testing,* ***QTS***: *quantitative tactile stimulation,* ***QVT***: *quantitative vibration threshold,* ***SD***: *standard deviation,* ***SNAP***: *sensory nerve action potential,* ***SSR***: *sympathetic skin response,* ***TCSS***: *Toronto Clinical neuropathy Scoring System,* ***TDT***: *thermal discrimination threshold,* ***TRI***: *thermal recovery index,* ***VPT***: *vibration perception threshold,* ***WHO***: *World Health Organization.* **†** *Method of assessment not reported,* **‡** *definition of neuropathy not reported,* **§** *definition of prediabetes not reported.*
